# Editorial: Blood-Based Biomarkers in Acute Ischemic Stroke and Hemorrhagic Stroke

**DOI:** 10.3389/fneur.2022.866166

**Published:** 2022-02-24

**Authors:** Timo Uphaus, Heinrich J. Audebert, Michael W. Graner, Steffen Tiedt, Robert G. Kowalski

**Affiliations:** ^1^Department of Neurology, Focus Program Translational Neuroscience, Rhine Main Neuroscience Network, University Medical Center of the Johannes Gutenberg University Mainz, Mainz, Germany; ^2^Center for Stroke Research Berlin and Department of Neurology, Charité - Universitätsmedizin Berlin, Berlin, Germany; ^3^Department of Neurosurgery, Anschutz Medical Campus, University of Colorado, Aurora, CO, United States; ^4^Institute for Stroke and Dementia Research, University Hospital, LMU Munich, Munich, Germany; ^5^Department of Neurology, Anschutz Medical Campus, University of Colorado, Aurora, CO, United States

**Keywords:** ischemic stroke (IS), hemorrhagic stroke, differential diagnosis, stroke etiology and classification, prognosis

Stroke is one of the leading causes of death and disability worldwide (1). The application of acute treatment strategies is limited by several factors such as a narrow time window of reperfusion treatments in ischemic stroke as well as an incomplete understanding of biologic mechanisms of secondary brain damage; blood-based biomarkers might inform on local and systemic pathophysiological processes, assist in patient selection for treatments, and thus support clinical decision-making in the acute phase.

Emerging reperfusion therapies in acute ischemic stroke (AIS) as well as evolving strategies to reverse anticoagulation in hemorrhagic stroke require early differentiation of stroke type. Frequently used emergency imaging such as computed tomography (CT) distinguishes ischemic and hemorrhagic stroke and thereby opens the avenue for recanalization therapies if intracranial hematoma is absent. Nonetheless, such imaging techniques are typically unavailable in the prehospital setting; CT-scans are also not sensitive to show ischemic brain lesions in the hyperacute setting. Experimental investigations proposed microRNA (miRNA) and exosomes as well as metabolites as markers of cerebral ischemia that might support AIS diagnosis. However, translational data in humans are sparse.

Prognostication after stroke depends on the interplay between demographic factors (e.g., age, sex, ethnicity), stroke subtype, and stroke etiology, as well as clinical severity. Persisting disability following ischemic stroke is a result of neuronal death, network dysfunction, and synaptic loss; specific markers of neuronal damage showed superiority over others in predicting functional outcome after stroke (2, 3), whereas cardiac markers indicating comorbidities such as atrial fibrillation showed only utility in prediction of mortality (4). Moreover, a first ischemic stroke is associated with increased risk of further cerebrovascular and other events, calling for the unraveling of stroke etiology in order to select patient-dependent appropriate secondary preventive medication. The additional consideration of biological information through blood biomarkers might improve the prognostic assessment as well as etiologic work-up, as compared to routinely available information, mainly based on purely clinical and imaging information.

Overall, there is a clear need for experimental as well as translational research on blood-based molecular and cellular biomarkers for differentiation of stroke and stroke mimics, stroke types (AIS vs. hemorrhagic stroke), to guide individual treatment decisions and provide information to patients and relatives. The same applies for the determination of stroke etiology and for the better understanding of secondary neuronal damage in order to develop new treatment paradigms within the hyperacute phase of AIS and hemorrhagic stroke.

## microRNAs

microRNAs are small non-coding RNAs with a length of ~22 nucleotides that regulate gene expression by destabilizing and repression translation of complementary mRNAs. They are characterized by a high expression in mammalian brains and are involved in modulation of excitotoxicity, microglia polarization, oxidative stress, neuronal apoptosis, and oxidative stress, all together mechanisms orchestrating secondary brain damage and thereby regulating functional recovery following AIS (5, 6). There is growing evidence from animal models that miRNA-based treatments with enhancers and inhibitors are able to penetrate the blood-brain barrier (BBB) using specific carriers (e.g., exosomes, liposomes, and lentiviruses) (7) and beneficially modulate brain ischemia. They might therefore be incorporated in novel therapeutic strategies to improve functional outcome in stroke patients by targeting detrimental mechanisms in the hyperacute and subacute phase of stroke.

From a diagnostic point of view, an increasing number of studies have identified stroke-specific patterns of circulating miRNAs that were also associated with symptom severity as well as infarct volume and predicted functional outcome. Patients with AIS showed higher circulating levels of miR-125a-5p, miR-125b-5p, and miRNA-143-3p compared to patients with transient ischemic attack (TIA) or neurologically normal subjects with a return to control levels 2 days after symptom onset, supporting the utility of these miRNAs for early stroke diagnosis in the emergency setting (8). Furthermore, several miRNAs may help in providing prognostic information for consultations with patients and relatives; and guide treatment decisions, as they are associated with functional outcome and mortality following stroke (9, 10).

In a translational approach, Cepparulo et al. compared miRNA levels between animal models of ischemic stroke (middle cerebral artery occlusion—MCAO) and hemorrhagic stroke (collagenase-induced hemorrhagic stroke). They demonstrated upregulation of specific miRNAs as early as 3 h after the procedure in these distinct animal models, pointing toward a potential clinical usage in differentiating ischemic from hemorrhagic stroke in the clinical setting.

## Omic-Approach

Besides miRNA, “Omics” reflecting specific pathophysiologic aspects of ischemic as well as hemorrhagic stroke have been investigated as potential biomarkers for stroke diagnosis, differentiation of ischemic vs. hemorrhagic stroke, prediction of functional outcome, and risk of stroke recurrence.

Within this Topic Section collection of articles, Malicek et al. aimed to unravel potential new candidates for differentiation of ischemic and hemorrhagic stroke by using an exploratory proteomic-based pilot study. They identified nine potential candidates connected with the immune system, the coagulation cascade and apoptotic processes. These markers now have to be validated in larger cohorts during the hyperacute phase and with additional analysis such as ELISA, Western Blot, and Mass spectrometry. These efforts aim to shorten treatment delay in stroke patients eligible for recanalization therapies by enabling prehospital differentiation of stroke subtypes.

In order to improve individualized stroke treatment, biomarkers enabling assessment of patients' specific stroke outcome are needed. In this article collection on blood-based biomarkers of acute ischemic and hemorrhagic stroke (11), the usefulness of inflammatory markers (Kirzinger et al.; Li et al.; Sun et al.), markers for oxidative stress (Kuwashiro et al.), vasoactive peptides (Westphal et al.), and BBB function (Müller et al.) are investigated. In addition to these markers reflecting specific pathophysiologic aspects of ischemic/hemorrhagic stroke, individual patient outcome is additionally determined by stroke-associated complications such as infections (Gens et al.; Zhang et al.) and cardiac comorbidities (Lin et al.). Beside their usefulness in predicting stroke prognosis, markers of cardiac pathology might also be useful in uncovering stroke etiology, as it is reported for natriuretic peptides and their role in identifying patients with atrial fibrillation (12). In addition, by taking advantage of coagulation cascade assessment via D-Dimer, risk of in-hospital mortality could be estimated in stroke patients with concomitant COVID-19 infections as demonstrated by Kim et al.

Patients suffering from stroke experience a higher rate of stroke recurrence. Identifying patients with increased risk of stroke recurrence might help to improve secondary preventive strategies and to select patients for intensified stroke etiology work-up and prevention support programs. Pable Hervella et al. identified soluble tumor necrosis factor-like inducer of apoptosis (sTWEAK) as a marker for endothelial dysfunction, to be associated with stroke recurrence and progression of cerebral white matter lesions.

## Exosomes

Exosomes (30–150 nm) belong to the family of extracellular vesicles, together with shedding microvesicles (or ectosomes 10–1,000 nm) and apopototic bodies (50–5,000 nm). Emerging evidence underlines a potential role of exosomes as diagnostic, therapeutic, and prognostic marker in stroke. Exosomes are endosome-derived vesicles; the following steps describe their formation: initiation, endocytosis, multivesicular body formation, and exosome secretion. Exosome secretion means the final process, in which the previously formed multivesicular bodies are fused with the plasma membrane and are finally secreted by their cell of origin. As the intracellular origin of secreted exosomes is seldom demonstrated, the term “small extracellular vesicle” may be more accurate (13), but “exosome” is nonetheless frequently used (14). After release, exosomes can interact with recipient cells by different biochemical processes, such as endocytosis, fusion, and ligand–receptor interaction (15).

Due to the fact that exosomes are abundantly secreted by most human cells, they play an exceptional role in intercellular signaling via cell-to-cell communication. Moreover, they are characterized by a lipid bilayer with an aqueous core, thereby harboring the ability to cross the BBB and transport various molecules across the BBB (16). This feature represents a prerequisite for both, transport of potential treatment to the target site within the CNS, as well as transport of markers mimicking pathophysiological CNS processes from CNS compartments to extra-CNS compartment (e.g., blood stream), where they can be easily accessed by venous puncture. Altogether, exosomes are a promising way of enabling the interaction between components of the neurovascular unit (neurons, glial cells, brain vessels).

With this in mind, exosome-derived markers might be a powerful tool for distinction of stroke subtypes and obtaining information on the pathophysiologic state within the CNS. So far, multiple exosome-derived miRNAs were associated with ischemic stroke outcome [e.g., miRNA-223 (17), miRNA-134 (18)] and might be able to identify patients who could benefit from reperfusion therapies in the hyperacute phase of ischemic stroke (19), as well as differentiate hemorrhagic from ischemic stroke patients (20). Exosomes are therefore a promising strategy to exclude hemorrhagic stroke and enable timely application of reperfusion therapies especially intravenous thrombolysis in the prehospital setting of AIS. Nonetheless, exosomes are more extensively studied as potential therapeutics (21), compared to approaches to use them as diagnostic tools, at least in the area of stroke pathology.

Within the current article collection, Jödicke et al. analyzed dynamic changes of extracellular vesicles in the biomarker and perfusion-training-induced changes after stroke (BAPTISe) study and uncovered an association with functional outcome in patients with subacute stroke.

## Summary

The current Research Topic, “Blood-Based Biomarkers in Acute Ischemic Stroke and Hemorrhagic Stroke” (11) provides a collection of articles that identify and validate blood-based biomarkers that deepen our understanding of stroke pathophysiology and that might support clinical decision-making for patients with ischemic as well as hemorrhagic stroke in the future.

## Author Contributions

TU, HA, MG, ST, and RK edited the Research Topic and drafted and revised the editorial manuscript. All authors have read and approved the final version of the manuscript.

## Conflict of Interest

The authors declare that the research was conducted in the absence of any commercial or financial relationships that could be construed as a potential conflict of interest.

## Publisher's Note

All claims expressed in this article are solely those of the authors and do not necessarily represent those of their affiliated organizations, or those of the publisher, the editors and the reviewers. Any product that may be evaluated in this article, or claim that may be made by its manufacturer, is not guaranteed or endorsed by the publisher.
